# Drivers’ Attention Strategies before Eyes-off-Road in Different Traffic Scenarios: Adaptation and Anticipation

**DOI:** 10.3390/ijerph18073716

**Published:** 2021-04-02

**Authors:** Zhuofan Liu, Wei Yuan, Yong Ma

**Affiliations:** 1 Modern Postal College, Xi’an University of Posts & Telecommunications, Xi’an 710121, China; 2 School of Automobile, Chang’an University, Xi’an 710064, China; yuanwei@chd.edu.cn; 3 Key Laboratory for Automotive Transportation Safety Enhancement Technology of the Ministry of Communication, PRC, Chang’an University, Xi’an 710064, China; mayong@chd.edu.cn

**Keywords:** visual attention, attention distribution, visual occlusion, adaptation, anticipation, driving simulator

## Abstract

The distribution of drivers’ visual attention prior to diverting focus from the driving task is critical for safety. The object of this study is to investigate drivers’ attention strategy before they occlude their vision for different durations under different driving scenarios. A total of 3 (scenarios) × 3 (durations) within-subjects design was applied. Twenty-three participants completed three durations of occlusion (0, 1, and 2 s) test drive in a motion-based driving simulator under three scenarios (urban, rural, motorway). Drivers’ occlusion behaviour, driving behaviour, and visual behaviour in 6 s before occlusion was analyzed and compared. The results showed that drivers tended to slow down and increased their attention on driving task to keep safety in occlusion 2 s condition. The distribution of attention differed among different driving scenarios and occlusion durations. More attention was directed to *Forward* position and *Speedometer* in occlusion conditions, and a strong shift in attention from *Forward* position to *Road users* and *Speedometer* was found in occlusion 2 s condition. *Road users* was glanced more frequently in urban road with a higher percentage of attention transitions from *Forward* position to *Road users*. While gaze switching to *Speedometer* with a higher intensity was found on motorway. It suggests that drivers could adapt their visual attention to driving demand and anticipate the development of upcoming situations by sampling enough driving-related information before eyes-off-road. Moreover, the adaptation and anticipation are in accordance with driving situation and expected eyes-off-road duration. Better knowledge about attentional strategies before attention away from road contributes to more efficient and safe interaction with additional tasks.

## 1. Introduction

With the increased presence of in-vehicle information systems, it becomes more common that drivers engage in a variety of non-driving-related activities while driving. Interaction with secondary tasks such as navigation, watching videos, and chatting/texting on mobile phones always leads to visual distraction. Visual distraction is related to driving with visual attention away from the road, which has long been known to contribute to crashes [1]. The potential for a particular visual distraction to increase crash risk depends on the process of secondary task engagement and disengagement [2]. It means when, where, how long and how often to sample information plays a core role in driving safety.

It is reported that only a small rate of crucial incidents occurred compared with the vast majority of safe engagement in visually distracting activities in natural driving [3], and driving with secondary tasks in daily life was not perceived dangerous from the perspective of most drivers [4]. Driving is a partial self-paced and satisficing task, which means drivers are able to make deliberate decisions whether they want to be distracted or not in a given situation. The decisions were made depending on the expectation of the future development of a situation and the perceived demand of driving tasks. If drivers could anticipate the development of potential dangers and apply situational-adaptive visual behaviour to sample enough necessary information to meet driving demand, they were still regarded as being attentive whether there was interaction with secondary tasks or not [5].

Anticipation is therefore a major factor in driving. It was found that drivers employ anticipatory strategies by planning when and where to make a call [6]. The analysis of occlusion behaviour showed that occlusion probability varied across driving routes, which demonstrated that drivers anticipate the development of upcoming situations and chose special locations for initiating occlusion tasks in accordance with their perception of current driving demand [7]. Metz proposed a cognitive approach to describe the attention process while driving with additional visual tasks [8]. It is assumed that attentional control during distraction is a mental state model based on driving. Before engaging in an additional secondary task, drivers should assess the driving situation to decide if it is possible to attend to the distracting activities or not. When drivers predict the driving situation in the near future will not be demanding and critical incidents will not occur, they may decide to conduct secondary tasks. 

However, drivers can also be distracted by events that are not planned, such as incoming phone calls, messages, or passenger interactions. In this case, drivers need to adjust their behaviour to keep safety. It is reported that drivers have the potential to adapt to distracting activities according to situational demands [9]. Numerous researchers proposed that drivers try to compensate for the reduced attention resource to driving tasks by reducing speed or even stopping the vehicle [10] and apply situational-adaptive visual behaviour to pick up necessary driving-related information [11]. Consistent with those results, it was found that drivers usually employ compensation strategies to deal with additional driving tasks in a simulator study [12]. In addition, a test track experiment showed that drivers adjusted time-sharing behaviour according to driving demands when they were distracted [13].

Although anticipation and behavioural adaptation theory in driving can explain drivers’ dynamic strategies of interacting with secondary tasks to maintain driving safety, much less is known about the dynamic distribution of visual attention before initiating additional tasks in different driving scenarios. Anticipation and adaptation in driving are cognitive processes that include the perception of characteristic cues and predicting the upcoming changes in traffic, which is mainly controlled by the visual system. A limited number of studies suggest that drivers fixed their attention on a smaller part of the visual field during the decision stage prior to initiating secondary tasks compared to normal driving without distraction [8]. However, eye movement behaviour can not reflect which targets should drivers notice and how long they need to obtain this information. Divers continuously or consecutively direct their gaze to various areas to pick up the important driving-related information based on their cognitive processing of the situation [14]. When and where to look depends much on both sub-driving-goal (top-down mechanism referring to attention is guided based on prior knowledge and current goals) and stimulus properties of an object (bottom-up mechanism means attentional guidance is purely driven by external traffic information) [15]. 

Lots of researches demonstrate that driving context could influence drivers’ glance behaviour [16,17]. For example, it is required to look at pedestrians, intersections, bus stops, traffic lights, parked cars, cyclists, and other surrounding traffic frequently when driving on an urban road. While, driving on a motorway only requires drivers to update information about driving speed, surrounding traffic, and lane position. The frequency and duration of information sampling are related to the time duration since the information was sampled from last time and the innate characteristics of the contextual variables [18]. In high demand situations which are difficult to anticipate, drivers usually direct more attention to the driving-related information to update the mental model with new information [19].

The distribution of visual attention is not only influenced by the driving scenario as mentioned above but also influenced by the driving task [20]. When driving with additional visual tasks, both the main driving task and the additional task compete for visual attention. Drivers are seen as active coordinators of driving demand and attentional resources. They could actively distribute their attention between driving and the distracting tasks and decide when to start or attend to additional tasks [21]. If the secondary task is highly demanding such as video calling which requires long eyes-off road and difficult to interrupt, drivers may reject or delay it, but if such task was much motivated by drivers such as operating navigation, they need to sample traffic-related information longer to develop a more sufficient mental model. The model could reflect both current and future driving situations before they initiate an additional task. As longer intensive sampling help drivers get more detailed information, thus they could predict the development trend of traffic in the near future better. The mental model should bear the accumulated uncertainty and meet the required situation awareness [22]. It is regarded that drivers’ uncertainty accumulates over time with their visual attention away from road. Before the uncertainty reaches one’s tolerance level, the driver will switch attention back to the road to reduce the discomfort of high uncertainty. If the driver continues directing their gaze away from the road after uncertainty exceeds the tolerant level, it will take more effort to acquire the situation awareness when they switch attention back to road [23]. On the other hand, drivers gradually become less aware of the driving situation over time with the eyes-off-road duration increasing [24]. The longer eyes-off-road duration, the more awareness of dynamic driving context diminishes, which is associated with lower driving performance and higher risk of safety-critical incidents [2]. When off-road glances exceeded 2 s, more frequent lane deviation and slower response to lead vehicle braking were observed [25]. Therefore, it is assumed that drivers may apply different visual strategies in response to different eyes-off-road durations.

Visual occlusion and eye-tracking techniques have been demonstrated as effective measures to study driver’s attention resource allocation [26,27]. Visual occlusion involves the blocking of drivers’ visual field intermittently by blanking the display or occlusion glasses in visual demand study [28]. It could be used to set accurate eyes-off-road duration. Eye-tracking has been used to study which and how often visual information is taken in, and how do drivers sample this information. In combination with occlusion and eye-tracking, it is possible to quantify drivers’ distribution of visual attention before diverting attention away from road for different durations.

In summary, these insights seem to suggest that drivers would sample relevant information to keep a good mental representation of the driving situation including current and future development of the situation in advance of conducting secondary tasks. Both driving scenarios and secondary tasks would affect the distribution of attention. However, where and how do drivers sample necessary information before different duration of eyes-off-road has not been systematically investigated yet. In addition, the understanding of drivers’ visual attention distribution before conducting secondary tasks in different driving scenarios is limited. Therefore, the purpose of this study was to examine drivers’ attention strategies during 6 s before different eyes-off-road durations in three different driving scenarios (urban road, rural road, motorway). More specifically, the following questions were addressed:

- Where do drivers look in preparation for different durations of eyes-off-road in different driving scenarios?
- How much attention do drivers spend for sampling different target information prior to occluding vision in different driving scenarios?
- What are the drivers’ visual search patterns in terms of attention transition before vision occlusion?

## 2. Materials and Methods 

The research was conducted in a driving simulator to avoid serious incidents on the real road, as the long eye-off-road duration maybe not safe in real driving. 

### 2.1. Participants

Participants were recruited from a stable database of participants and through personal contact. They were in good mental and physical health. Additional recruitment criteria were that they all have a good driving record and no motion sickness. The final sample consisted of 26 participants (10 female) between the ages of 22–45 years (M = 31.4; SD = 5.7 years) with a driving experience of 6–13 years (M = 8.4; SD = 2.5). All participants received 200 RMB as compensation for their efforts.

The study was conducted according to the guidelines of the Declaration of Helsinki, and approved by the Institutional Review Board of Chang’an University (protocol code 2018/0105-01). Informed consent was obtained from all subjects involved in the study.

### 2.2. Apparatus

**Figure 1:**
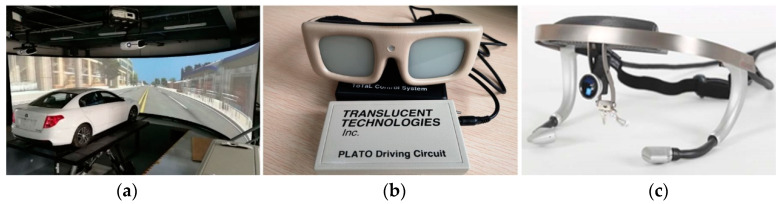
Experimental apparatuses in the experiment. (**a**) Driving simulator; (**b**) PLATO goggle; (**c**) Dikablis eye- tracker.

The experiment was carried out in a motion base driving simulator with 6 degrees of freedom (Figure 1a). It was equipped with an automatic gearbox. The cabin is modified by the original car Besturn B50 with a high-precision vehicle dynamic simulation model. The projection of the driving scene is realized with a high-definition ring screen displaying a 180 × 30° field of view in front of the vehicle.

PLATO (Portable Liquid crystal Apparatus for Tachistoscopic Occlusion) goggle was used to set accurate eyes-off-road duration (Figure 1b). This is a kind of semi-self-paced vision occlusion method, which means participants could occlude the front view for a pre-defined duration. After a certain duration, the goggle would open again. The goggle was always open unless participants press the control button which was attached to their finger, and the experimenter would tell them the occlusion duration before the start of the next driving condition. The Dikablis eye tracker that was worn on participants’ heads was used to record eye movements. It was consisting of a small front camera and two built-in cameras (Figure 1c). The two built-in cameras record eye movements with 60 Hz and display them superimposed on a scene view video recorded by the front camera.

### 2.3. Experiment Design and Driving Task

**Figure 2:**
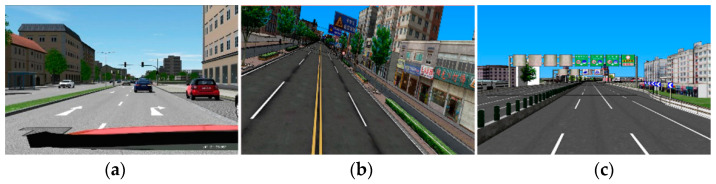
The three simulated environments. (**a**) urban road; (**b**) rural road; (**c**) motorway.

A test course of about 50 min was created consisting of urban road, rural road, and motorway (Figure 2). The three driving scenarios have different attentional demands on the driver. The posted speed limit for the three types of road was 60, 90, and 120 km/h. The urban road is relatively the most demanding, considering the existence of pedestrians, dense oncoming vehicles, intersections, traffic lights, cyclists, bus stops, and so on. On the contrary, the motorway was regarded as the least demanding scenario, as only discrete oncoming vehicles, lead vehicle, and gentle curves exist in the motorway. While diving in rural area implied a moderate demand with the presence of moderate oncoming vehicles, sharp curves and narrow lanes.

This study employed a 3 (driving scenario) × 3 (eyes-off-road duration) within-subjects design. The three types of driving scenarios (urban road, rural road, and motorway) were crossed with the three durations of eyes-off-road condition (0, 1, and 2 s), resulting in nine combinations, henceforth called sub-trails. Thus, each participant conducted 9 sub-trails. The order of appearance of 9 sub-trails was basically the same across participants to avoid learning effect, and they were set for each participant before the experiment. Each sub-trial was about 5 min long, and there was a 200 m neutral phase connecting each sub-trial. The neutral phase was not included in the analysis. 

### 2.4. Procedure

Upon arrival at the simulator hall, each participant read and signed an informed consent form. Then, an experimenter described the experimental trials and gave instructions on the driving tasks. After that, the participant sat in the simulator, wore PLATO goggles with a micro-switch for blocking the vision, and wore a head-mounted eye tracker, which was calibrated via 3-point-calibration. It is worth noting that wearing both the Plato goggles and the eye tracker at the same time is a bit inconvenient for the participants. It is required to tighten both of them to make sure they do not interfere with each other during the experiment. Therefore, the experimenter needs to be patient to adjust the eye camera and calibrate the eye tracker when participants wear both the Plato goggles and the Dikablis eye tracker. Then the participants practiced the operation of the driving simulator to acclimate to the simulator, three types of driving scenarios, and different durations of occlusion. After about 5 min of practice, participants were asked to drive at the speed limit posted in each scenario and try to close the goggle as often as possible when they have good situational awareness. However, they need to obey traffic rules and drive safely in the first place.

### 2.5. Analysis

The final dataset contains 23 participants. As the detection of eye-tracking quality was not reliable, data from three participants were excluded. Drivers’ visual behaviour, occlusion behaviour, and vehicle movement were recorded separately, but they were synchronized together base on logging time. 

The objective data of visual behaviour was only analysed for the period of 6 s before occlusion to investigate what is drivers’ visual attention strategy to prepare “enough” driving-relevant information before different durations of occlusion in different driving scenarios. It is reported that only glances in 6 s could reflect driving state [29]. First, the 6 s observation sequences were extracted from 1s occlusion condition and 2s occlusion based on the number of occlusions and the occlusion location. As a result, a total of 2200, 1400 6-s sequences were extracted in occlusion 1 s conditions and occlusion 2 s conditions separately. Second, the position of these 6 s observation sequences was located based on the coordinates in the scenarios. Third, the repeat occlusion sequences were deleted if the start points of occlusion were close to that in the existing occlusion. Fourth, the corresponding segments from the baseline were extracted based on the start position of occlusion, and 3300 6-s observation sequences were extracted from baseline. Next, all the 6 s observation sequences were sorted out according to driving scenarios. As a result, a total of 1680, 1900, and 2700 6-s sequences were extracted for urban road, rural road, and motorway, respectively. 

Frame by frame analysis was used to encode drivers’ eye glance behaviour. The video stream that contained eye movement information was imported to a video player, and two experienced researchers coded the glance location and duration manually. Then, a third researcher inspected the results to guarantee the coding quality. Drivers’ visual field was divided into five areas of interest (AOIs): “Forward”, “Road users”, “Speedometer”, “Mirror”, and “Others”. In addition, one occlusion case was also included when the 6 s sequence contained occlusions. Notably, “Road users” refers to other road users such as oncoming vehicles, pedestrians, and parked cars. “Others” includes areas not directly related to current driving. “Occlusion” is a result of a previous occlusion, which occurs when the interval of two occlusions is shorter than 6 s. Apparently, there was no occlusion in the baseline. The value of each AOI was set individually per participant to make sure that all the AOI could locate participants’ glance location accurately. Eye movements were analysed in accordance with the ISO-metrics number of glances and glance duration [30]. Three different indicators of visual behaviour were defined based on the encoded glance sequence, which were explained in Table 1. The effects of occlusion duration and driving scenario on occlusion behaviour, driving behaviour, and visual behaviour were computed using a 2-way repeated-measures analysis of variance. The significance level was set at α = 0.05.

The transition probability from location *i* to location *j* was calculated as the number of glances from location *i* to location *j N*(*i, j*) divided by the total number of glance transitions out of location *i N*(*i*).

$$p\left(i,j\right)=\frac{N\left(i,j\right)}{N\left(i\right)}$$

Here, the total transition probabilities of leaving one AOI to all AOIs sum up to one $\underset{i,j}{\sum }p\left(i,\;j\right)=1,i=1,\;2,\;3,\;4,\;5,\;6$. Smaller p(i,j) suggests a lower probability of the transition from location *i* to location *j*. The transition probabilities indicate the glance transition preferences before different eyes-off road duration. Basically, there is no self-transition, as glance transition was used here, but if participants glanced at one AOI for 6-s, which means there were no glance transitions, then p(i, i)=1.

## 3. Results

### 3.1. Occlusion Behaviour

Occlusion number was first analysed to see the occlusion behaviour per scenario and occlusion duration. The ANOVA test showed that both driving scenario (F (2, 44) = 26.7, *p* < 0.05) and occlusion duration (F (1, 22) = 19.5, *p* < 0.05) have significant effects on the number of occlusions. No interaction effect was demonstrated. The number of occlusions decreased with the demanding of driving scenarios, and a fewer number of occlusions was observed in the occlusion 2 s condition (Table 2). That is participants occluded their vison fewer times on the urban road than that on the motorway. In addition, variations of the number of occlusions were larger for occlusion 1 s condition and on the motorway. 

### 3.2. Driving Behaviour

**Figure 3:**
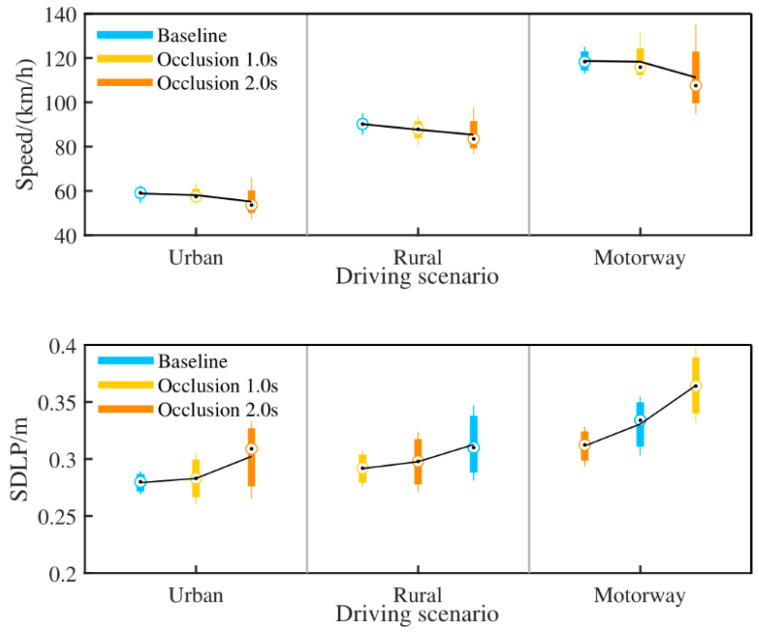
Boxplot of driving speed (**top**) and SDLP (**bottom**) per occlusion duration in scenario. For each box, the central mark is the median, and the mean values are lined with solid black lines.

Driving speed and standard deviation of lateral position (SDLP) were analysed to investigate longitudinal control and lateral control performance, respectively. Driving speed and SDLP per occlusion duration in different driving scenarios are illustrated in Figure 3.

The statistical tests found a significant effect of occlusion duration (F (2, 44) = 12.4, *p* < 0.01) on driving speed. It showed that driving speed was lower in occlusion 2 s condition, while there was no significant difference between the baseline and occlusion 1 s condition. Moreover, participants almost kept the limited speed in the baseline and occlusion 1 s condition. As for lateral control, both occlusion duration and driving scenario turned out to have a significant influence on SDLP. It was found that SDLP was significantly larger on motorway than that on urban road and rural road, and it increased with occlusion duration on motorway. While driving on urban road and rural road, no significant difference of the SDLP was found between baseline and occlusion 1 s condition. However, SDLP was much larger in occlusion 2 s condition. This indicates that the drivers direct visual attention away from the road for 2 s will impair both longitudinal and lateral control performance. Besides, lateral control performance will also decrease even drivers look away from the road just for 1 s when they drive fast.

Furthermore, lane exceedance was analysed in all driving scenarios and occlusion durations for investigation of driving error. The results showed that no vehicle crossing the lane was observed in all driving conditions. It indicates that all the participants did not lose control of vehicle when they direct their attention away from driving. 

### 3.3. Attention Distribution

Attention distribution was investigated by analysing attention ratio and number of glances to each of AOIs. As can be seen from Table 3, driving scenario and occlusion duration turned out to have a significant influence on almost all of the dependent variables, in four cases including an interaction effect between the two. 

**Figure 4:**
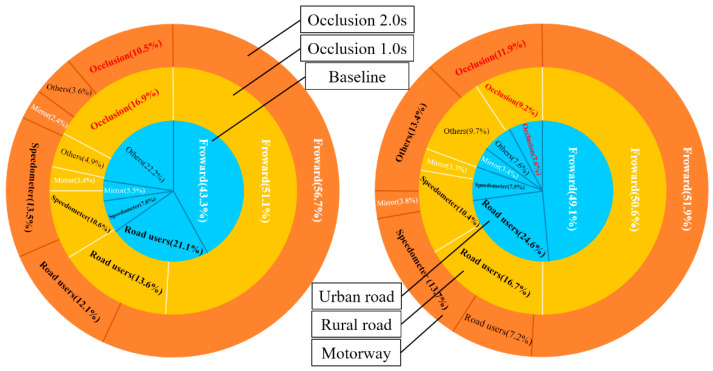
Attention ratio distribution as a function of occlusion duration (**left**) and driving scenario (**right**).

Attention ratio was computed by mean glance duration to the defined AOI divided by the analysed segment, which is shown in Figure 4. 

It was found that drivers were visually looking *Forward* areas at a proportion of more than 50% during occlusion conditions compared to 43.3% in the baseline driving where there was no occlusion. In contrast, *Road users* and *Others* received more attention in the baseline. Additionally, drivers reduced their attention to *Road users* and occluded less time in occlusion 2 s condition, but they directed more attention to the *Forward* and *Speedometer* in 6 s before the longer eyes-off-road. When driving in more demanding scenarios such as urban road, participants spent more time looking at *Road users* and less time on *Others*. Another observation was that there were still some amounts of *Occlusion* in the analysed period. Particularly, drivers occluded their vision more time that could be regarded as spare capacity in less demand scenarios. This arising spare capacity was also observed more in occlusion 1 s condition than that in occlusion 2 s condition. Meanwhile, with the increase of driving speed, *Speedometer* received more attention.

**Figure 5:**
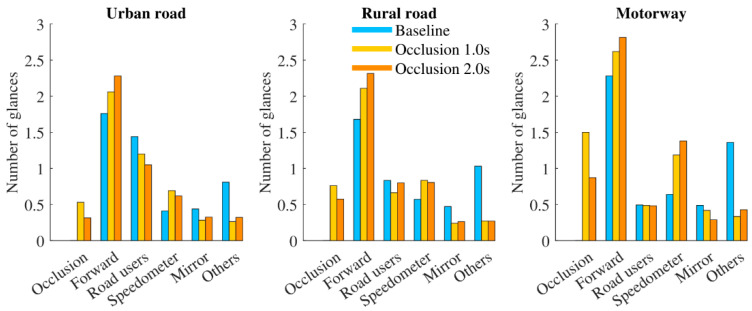
The number of glances towards each AOIs before eyes-off-road per road type and occlusion duration.

Histograms showing the total number of glances to different AOIs in three driving scenarios, divided by the different occlusion durations, are displayed in Figure 5. 

As presented in Figure 5, the distribution pattern of glance number to each AOI before occlusions among the three different scenarios were very similar. As expected, *Forward* and *Speedometer* were both glanced more frequently in the two occlusion conditions, and the number of glances looking at *Speedometer* was significantly higher in occlusion 2 s condition. In contrast, drivers reduced *Occlusion* times in occlusion 2 s condition. In addition, participants tended to glance at the *Road users* more frequently with the increasing complexity of driving scenario, especially in urban road, but this effect became less pronounced in the occlusion conditions. When driving in the motorway, gaze was directed more often to *Speedometer*. In combination with the analysis of attention ratio, it could be seen that the number of glances looking at the *Forward* was disproportionately higher in the motorway. It implied that glances duration to *Forward* area in the motorway was shorter than those in the urban and rural road.

### 3.4. Attention Transition

**Figure 6:**
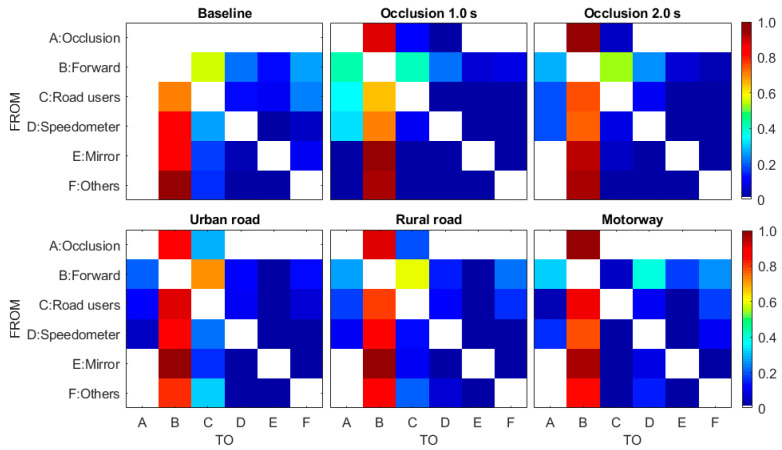
Glance transition graphs between the different AIOs. The colour indicates the probability of glance location moving from AOI Y to AOI X with warmer colour for greater value.

To shed further light on drivers’ attention strategy before occlusion, attention transition among different gaze targets was calculated to examine visual search patterns. Transition probabilities between different glance targets and occlusion are presented in Figure 6. Note that glance transition was calculated in this study, not the gaze transition, so there is no self-transition. 

Analyses of the attention transition matrix found that the main visual search pattern was featured by sequences of *Forward*–*non-forward area*–*Forward*. This pattern holds up for all conditions. This indicates that glance straight ahead is the default driving state. The three subfigures in the top row described participants’ visual attention transition preference for three different eyes-off-road durations. As expected, participants’ visual attention transitions during the two occlusion conditions were different from those in the baseline. It could be seen that participants shifted their attention among a broad number of regions including *Forward*, *Road users*, *Speedometer*, *Mirror*, and *Other areas* in the baseline. Specifically, attention transitions from other regions to *Forward* road are visualized with a higher intensity in the baseline. While gaze switching to *Mirror* and *Other* area from other targets is relatively rare in the two occlusion conditions. Another finding was that participants occluded front view more frequently after glancing at a broader number of regions in occlusion 1 s condition. While in occlusion 2 s condition, participants took significantly more attention transfer from *Forward* to *Road users* and *Speedometer*.

The three subfigures in the bottom row described the attention transition matrices for urban road, rural road, and motorway, respectively. Obviously, it showed a strong shift in attention from *Forward* to *Road users* on urban roads and rural roads, and gaze switch to *Road users* from a broader number of regions with higher intensity was found on urban road. In contrast, the spatial distribution pattern of attention on motorway illustrated that attention transitions from *Forward* to *Speedometer* occupied a high percentage. Another powerful result indicated that participants only occluded the front view after getting the information about the *Forward*, *Road users*, and *Speedometer*, and participants occluded the front view more times after glancing *Forward* on motorway.

## 4. Discussion

Previous analysis indicated that there was no big difference in the visual behaviour before and after occlusions in terms of number of glances to each target, percentage of dwell time, distribution of glance in the same scenario. Therefore, this research only focuses on drivers’ attention strategies of where and how they distribute their attention before conducting different difficulty of eyes-off-road visual tasks. This was achieved by blocking different durations of forward view while driving in three different simulated roads. The results show that drivers could adapt to the current driving demand and anticipate the upcoming driving situation. Moreover, they always prepare enough driving-related information before directing attention away from driving, but the attention strategy differs between different eyes-off-road durations and different scenarios.

The lower number of occlusions in highly demanding driving situations with longer eyes-off-road duration indicates that drivers perceived the driving demand and anticipate the potential demand that may be caused by visual distraction in advance of starting distracting activities. The lower number of occlusions also means longer time between two occlusions in the experiment, that is, drivers need longer time to glance sufficient information and cognitively process the more complex situations before occluding their vision. Moreover, the longer occlusion duration is associated with significantly increased uncertainty which is detrimental to safety. A longer time is required to build an adequate mental model to predict the development of the situation. Thus, participants take into account the situational demands and difficulty of occlusion task into their decisions for initiating occlusion task. It could be expected that drivers will not start a difficult distracting task in highly demanding driving situations in real driving. 

In driving performance, lower speed and higher SDLP in occlusion 2 s condition were found for longitudinal and lateral vehicle control, respectively. It provides further evidence that long time visual distraction impairs car-following and lane-keeping performance [31,32,33]. However, there was no lane exceedance occurring, which indicates that participants did not lose control of vehicle during the experiment. It could be expected that visual distraction for a short time does not affect driving safety and drivers could still have a good situation awareness. This can be shown by the good performance of longitudinal and lateral vehicle control in occlusion 1 s condition. These findings are in line with previous research indicating drivers tend to decrease driving speed and increase manoeuvre of the steering wheel when distracted in an attempt to avoid a collision with the leading vehicle and keep vehicle in lane boundary [10,34]. This adaptation behaviour can be understood as a form of self-regulation. In this way, they could anticipate the situational development and choose when and where to engage in secondary tasks.

Drivers’ visual behaviour is also adapted to the requirement of the driving situation. The urban environment is the most complex including parked cars, bus stops, traffic signs, and pedestrians, which requires drivers to glance at these variables for driving. Therefore, *Road users* was the most frequent glance targets except *Forward* area in urban road. Driving on motorway is least demanding in that fewer contextual variables and moderate interact traffic, but driving speed is the highest. Thus, there is no surprise that participants directed more attention with their gaze frequently to *Speedometer* before occlusions to control driving speed. Different from urban road and motorway, *Road users* and *Speedometer* received a certain amount of attention in rural road. It may be because the relative speed between ego vehicle and oncoming vehicles is much higher than that in urban road, and the road is narrow and curvy, which serves as a threat to drivers. Therefore, participants need to monitor the oncoming vehicle and speedometer frequently to keep driving speed and avoid a collision. 

It should be noted that there were still some *Occlusions* and *Others* before eyes-off-road, especially in the motorway. These *Occlusions* and *Others* could be regarded as part of visual spare capacity which varied with the demand of driving scenarios. In some way, the amount of *Occlusions* and *Others* could indicate spare capacity. As it is difficult to know with certainty how much spare capacity is contained in the “default” forward attention. Considering that motorway has the least demand for attention, participants have the largest amount of spare attention (11.9% *Occlusions* + 13.4% *Others*) in addition to observing the necessary traffic information, while there were only 7.6% *Occlusions* and 7.6% *Others* in urban road, which indicated that driver tried to spend more attention in observing the development of the situation to adapt to the demands of the situation. In some way, the spare attention before eyes-off-road can be interpreted as an indicator of a situation-aware use of secondary tasks in the vehicle.

In addition, drivers also seem to adapt their visual behaviour to anticipate the development of upcoming situations for the expected eyes-off-road duration. More attention was directed to *Forward* and *Speedometer* in occlusion conditions states that drivers focus their attention on special AOIs just before the eyes-off-road. It is speculated that drivers perceive traffic-related information based on their situation model, and they usually spend much attention on identifying important information to update the mental model. Moreover, participants glanced *Forward* and *Speedometer* more frequently in occlusion 2 s condition. This could be explained by the fact that drivers’ uncertainty increased significantly when eyes-off-road duration reached 2 s, as 2 s is relative long enough. That is why it has been set as one of the limiting criteria designing IVIS by the National Highway Traffic Safety Administration (NHTSA) [35], and participants try to get the driving-relevant information as detailed as possible and predict its development to reduce the expected upcoming uncertainty and maintain driving safety. 

From the analysis of attention transition, it is apparent that drivers employ different attention transition patterns before directing visual attention away from road when compared to baseline driving. Before eyes-off-road, more attention transfer from *Forward* to *Road users* and *Speedometer* indicates that drivers spend more time on getting information from the two areas because other road users and driving speed are two uncertain pieces of information that change constantly, and participants need to sample enough information to predict the trend of the two information in order to keep the mental situational model. Besides, attention transition patterns differ among different driving situations, featured by a high percentage of transitions from *Forward* to *Speedometer* on the motorway, while a strong shift in attention from *Forward* to *Road users* was found on the urban road and rural road, especially, gaze switches to *Road users* from a broader number of regions with higher intensity was found on the urban road. The findings imply that the driving scenario has a significant impact on attention transition, and it is consistent with previous studies that drivers looked more on perceived important targets during secondary tasks while driving [24]. Moreover, the result of attention transition is consistent with previous studies that drivers generally employed *Forward*–*non-forward area*–*Forward* search pattern. This indicates that drivers usually get information from windscreen. It is assumed that looking straight is the default state and glancing at other areas serves a purpose to sample the necessary information, as it deserves some efforts to turn head or shift eyesight. It could be inferred that drivers usually include the situational demands into their decisions for initiating occlusion task, and they could adapt their attention to the situational demands and search traffic-relevant information efficiently.

There are some limitations in this study that should be considered. First, the experiment was conducted in a driving simulator where no actual crash risk occurred. Participants may drive and occlude their vision aggressively. Therefore, the resulted distribution of attention may differ from the real road. Second, the occlusion method was employed to simulate drivers’ eyes-off-road durations, which is different from actual secondary driving tasks. During occlusions, participants’ minds may still be on driving without any additional cognitive load. Thus, it is unclear how far the results can be generalized. Third, the glance behaviour was only separately analysed by road types, not by the complexity of driving situation. Fourth, this study only focused on drivers’ attention strategy before they voluntarily diverted attention away from driving for less than 2 s. It may be interesting to know attention distribution change for interaction with unplanned secondary tasks or occlusion events that is longer than 2 s. These conditions may have yielded significantly different results and are representative of real-world conditions. Last but most important, the extracted 6 s before occlusion also contains the previous occlusion in this experiment, especially on the motorway, as some participants re-acquire situation awareness in less than 6 s after occlusion. This confounds the data of what is happening “before an occlusion” with the data that reflects “after an occlusion”. As recovery actions after occlusion have a particular impact on the drivers’ ability to react to events and avoid potential incidents due to the change in conditions transferring during the occlusion. Therefore, the results may mix drivers’ preparatory pattern of visual attention before occlusion and recovery pattern after occlusion together. It is difficult to know how much of the 6 s that reflects “before an occlusion”. It could be interesting to have longer intervals between occlusions, such that the attention strategies pre-occlusion becomes clearer. 

Future research should also include driving context variables to examine when, where, and how drivers engage in secondary tasks. As driving context influences drivers’ decision to engage in a visual-manual task, it could be expected that information decay speed and distance/time from ego-vehicle of driving context variables affect drivers’ attention distribution strategies. Before engaging in secondary tasks, the driver may pay more attention to targets with faster information decay speed and targets closer to the ego-vehicle in order to maintain good situational awareness. It would be interesting to investigate the preparatory glances in the surrounding traffic environment with different driving context variables. Moreover, it is strongly suggested that different types of daily secondary tasks should be used in the future to see how drivers plan to engage in different secondary tasks. If drivers only conduct certain secondary tasks in specific situations, it is interesting to investigate how drivers sample necessary information in the current situation before initiating secondary tasks. 

From an applied viewpoint, the results can be used for the development of human-cantered distraction detection to improve driving safety. Inexperienced drivers who like to engage in secondary tasks seem to need support to better share their attention across driving and secondary tasks by sampling enough necessary information before engaging in secondary tasks, and attention monitoring devices that provide warnings to drivers when they miss important information will no doubt aid this process. Moreover, the findings could support driver training and safety education programs with theoretical guidance.

## 5. Conclusions

In short, the results indicate that there was no significant evidence that engaging in easy self-initial occlusion tasks could influence driving safety in driving simulator. Concretely, drivers are able to adequately adapt their attention distribution to the current driving situation, and they try to sample enough driving-related information and anticipate the development of upcoming situations to create an adequate mental model before occluding their vision. Additionally, they take into account both situational demands and difficulty of occlusion tasks into their decisions for the interaction with the occlusion task. 

## Figures and Tables

**Table 1:** Description of motivation and analysis of the three indicators of visual behaviour in this study.

Indicator	Motivation	Analysis
number of glances	how often do the drivers look at each AOIs in 6 s	number of glances directed at each AOI in the analysed segment
Attention ratio	how much do the drivers look at each AOIs	the accumulated time that the participants looked at each AOI divided by the analysed duration
Attention transition	what are drivers’ visual search patterns in the spatial domain	driver’s attention location shifted from one AOI to another AOI

**Table 2:** Average number of occlusions per occlusion duration and driving scenario.

Driving Conditions	Average Number of Occlusions		
	Urban	Rural	Motorway
Occlusion 1 s	26.5 ± 7.2	30.2 ± 10.3	41.4 ± 16.3
Occlusion 2 s	16.3 ± 4.8	18.9 ± 6.9	27.6 ± 9.7

**Table 3:** F-values and levels of statistical significance for ANOVAs of attention distribution with the factors of occlusion duration and driving scenario in a full factorial design.

Attention Distribution	AOIs	Scenarios F (2, 44)	Durations F( 2, 44)	Scenarios * Durations F (4, 88)
Attention ratio	Occlusion	11.2 ***	F (1, 22) = 6.7 *	0.4
	Forward	7.4 **	1.2	4.9 *
	Road users	17.1 ***	11.4 **	8.2 *
	Speedometer	12.8 ***	8.4 **	10.1 **
	Mirror	0.8	0.5	0.5
	Others	8.7 *	11.9 **	0.7
Number of glances	Forward	1.9	8.1 **	0.7
	Road users	12.1 ***	20.4 ***	7.8 **
	Speedometer	10.3 **	13.9 **	1.2
	Mirror	0.7	0.2	0.4
	Others	10.4 *	7.7 *	0.4

Notes: * *p* < 0.05, ** *p* < 0.01, *** *p* < 0.001.

## Data Availability

The data presented in this study are available on request from the corresponding author.
